# Renin-angiotensin system as an emerging target to modulate adult neurogenesis in health and disease

**DOI:** 10.1186/s13287-025-04430-2

**Published:** 2025-07-01

**Authors:** Jannette Rodríguez-Pallares, Lucia A. Garcia-Crivaro, Juan A. Parga, Jose Luis Labandeira-Garcia

**Affiliations:** 1 Research Center for Molecular Medicine and Chronic Diseases (CiMUS), Health Research Institute of Santiago de Compostela (IDIS), Universidade de Santiago de Compostela, 15782 Santiago de Compostela, Spain; 2 Networking Research Center on Neurodegenerative Diseases (CIBERNED), Madrid, Spain

**Keywords:** Subependymal zone, Subgranular zone, Hippocampus, Angiotensin, AT1 receptors, AT2 receptors, Neurodegeneration, Neuroinflammation, Cognitive impairment, Aging

## Abstract

Adult neurogenesis is a complex multiphase process involving the formation and integration of new neurons into existing brain circuits. Although it was first described over 50 years ago and numerous factors involved in regulating neurogenic niches have been extensively studied, the underlying molecular mechanisms and interactions involved in controlling adult neurogenesis are still not fully understood. The renin-angiotensin system (RAS) is a well-known hormonal system that controls water and electrolyte balance and blood pressure. In addition to the circulating RAS, a local brain RAS has been described, with a key role in brain homeostasis. A wealth of evidence has emerged showing the involvement of RAS in neurodegeneration and neuroinflammation as well as in proliferation, differentiation, survival, and regeneration processes. Moreover, RAS has a role in cognitive function, behavioral responses, and dementia, which are closely related to neurogenic areas. This review summarizes the current evidence on the role of RAS in regulating adult neurogenic niches. We critically discuss pre-clinical and clinical studies investigating the role of RAS as a potential therapeutic target to modulate neurogenesis in pathological conditions.

**Graphical abstract:**

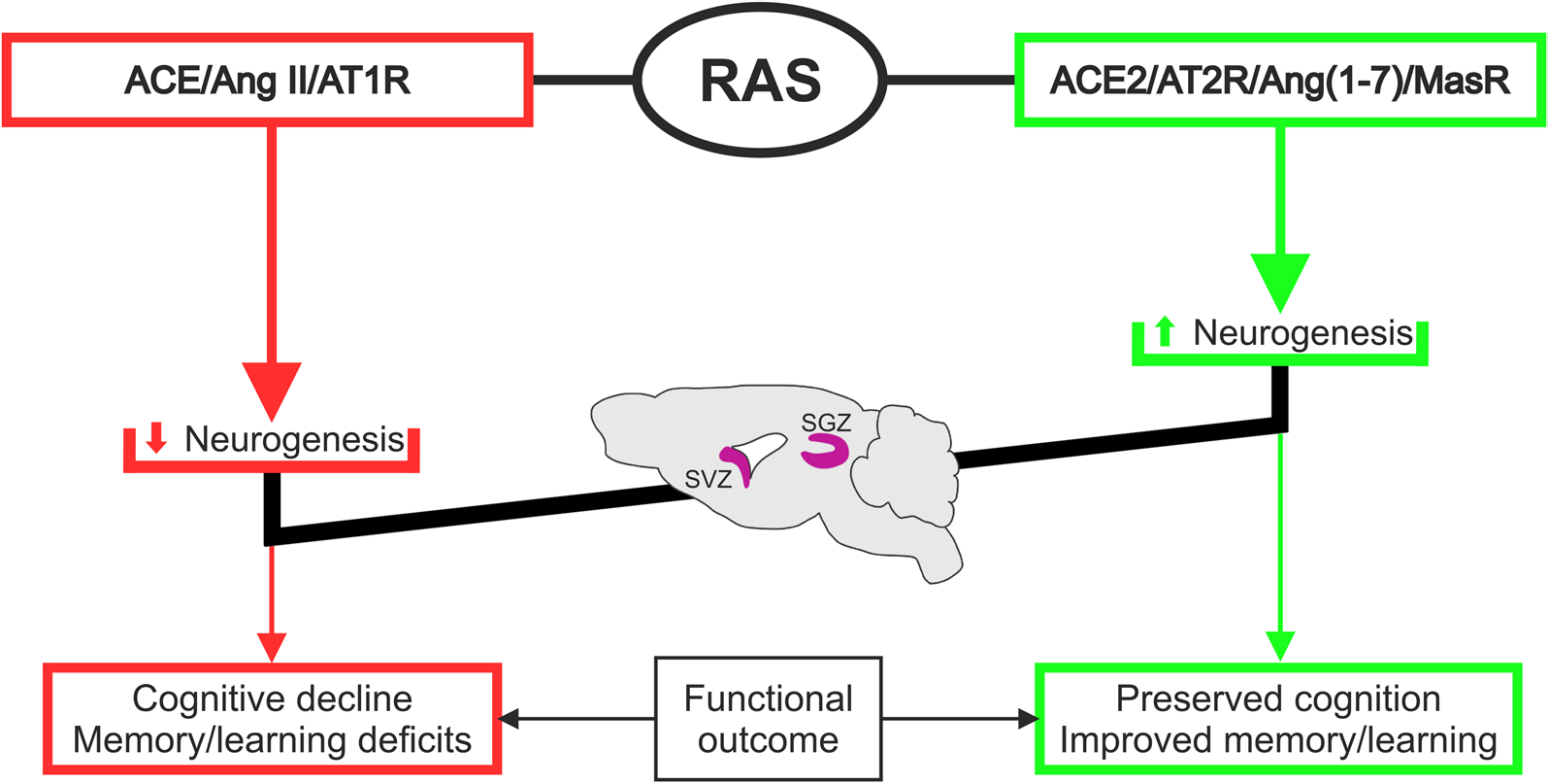

## Background

Neurogenesis is a multistage process that implies the formation of functional neurons in the brain and is underpinned by the existence of neural stem cells (NSCs), whose proliferation and differentiation are precisely regulated both spatially and temporally. Although the functional significance of adult neurogenesis is not well known, there is evidence of its contribution to homeostatic processes and neural plasticity, supporting learning, memory, and affective behaviors, among others [1]. Different evidence shows that neurogenesis is affected during aging or by brain insults or pathologies such as stroke or trauma. Alterations in neurogenesis also appear to be a common hallmark in neuropsychiatric or neurodegenerative diseases [2], and these alterations could contribute to the symptoms of the disease including cognitive decline and memory deficits. The factors that regulate adult neurogenesis have been broadly studied in healthy and disease conditions. However, a better characterization of the underlying mechanisms that fine-tune neurogenesis is essential to identify new targets for reversing the alterations in neurogenesis associated with pathological conditions or harnessing endogenous neurogenic potential as a possible therapeutic strategy for the regeneration of damaged areas [3].

There is much evidence of the involvement of the renin-angiotensin system (RAS) in neurodegeneration and neuroinflammation, and drugs targeting RAS, commonly used for the treatment of hypertension, showed neuroprotective effects [4, 5]. RAS also participates in processes of neuronal differentiation, survival, and regeneration [6, 7], and there is evidence of the role of RAS in cognitive function, behavioral responses, and dementia, which are closely related to neurogenic areas [8]. Taking this dual role of RAS, involved both in central nervous system dysfunction and neuroprotective/growth-promoting actions, we will compile the existing evidence on the role of RAS as a new protagonist in the regulation of adult neurogenesis both in physiological and pathological conditions.

### Neurogenic niches in the adult brain

**Fig. 1:**
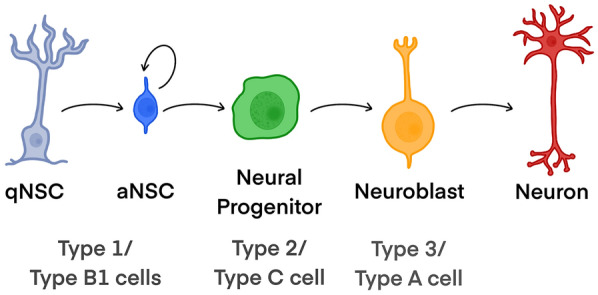
Schematic representation of cell lineage progression in adult neurogenesis in the ventricular-subventricular zone (V-SVZ) and dentate gyrus (DG). Quiescent neural stem cells (qNSC) give rise to activated NSCs (aNSC) (type 1 cells in the DG and type B1 cells in the V-SVZ), which first transit to highly proliferative progenitor cells (type 2 cells in the DG and type C cells in the V-SVZ) and then to neuroblasts (type 3 cells in the DG and type A cells in the V-SVZ), which mature into neurons

Adult neurogenesis occurs throughout life in two well-established or ‘canonical’ brain regions in mammals: the subgranular zone (SGZ) and the ventricular-subventricular zone (V-SVZ). The SGZ is a thin germinal layer in the hippocampal dentate gyrus (DG). This niche contains NSCs or radial-glia-like cells, also called type 1 cells [9, 10], that express astrocytic markers such as glial fibrillary acidic protein (GFAP) and other NSC markers such as nestin or Sox2. Activated radial-glia-like cells can divide to self-renew or give rise to intermediate progenitor cells, also known as type 2 cells, with transit amplifying characteristics and positive for nestin. These cells give rise to type 3 cells or neuroblasts, positive for doublecortin (DCX) and the polysialylated neural-cell-adhesion molecule (PSA-NCAM), which mature to become excitatory neurons that express calbindin and fully integrate into the circuitry [11]. This niche also contains other cell types that support neurogenesis, such as microglial cells, non-neurogenic astrocytes, and a dense vascular network [12]. On the other hand, the V-SVZ is the largest germinal zone in the adult brain. This niche, lining the wall of the lateral ventricles, also contains GFAP-positive NSCs (type B1 cells) with radial glial properties [13]. Activated B1 cells give rise to transit-amplifying progenitors (type C cells), that express Mash1 (Ascl1) and Dlx2 among others. C cells divide a few times before generating neuroblasts (type A cells), positive for DCX and PSA-NCAM, that migrate tangentially through the rostral migratory stream to the olfactory bulb. When neuroblasts reach the olfactory bulb, young neurons ultimately differentiate into different subtypes of interneurons that integrate into the circuitry [10] (Fig. 1).

### Factors regulating adult neurogenesis

Different key factors and signaling mechanisms interact and tightly regulate adult neurogenesis ensuring neuronal turnover [14]. Intrinsic factors include transcription factors such as Sox2, Pax6, epigenetic control, and non-coding RNAs [15]. Notch signaling is pivotal in NSC maintenance and differentiation, with context-dependent effects. Sonic hedgehog plays an important role in the formation and patterning of adult neurogenic niches. Wnt signaling is critical in regulating NSC behavior, controlling self-renewal, proliferation, differentiation, and functional integration of newly formed neurons. Numerous neurotrophic and growth factors such as brain-derived neurotrophic factor (BDNF), vascular endothelial growth factor (VEGF), fibroblast growth factor (FGF)−2, epidermal growth factor (EGF), or insulin growth factor (IGF)−1 are involved in NSC proliferation and generation of new neurons. Similarly, different cytokines, including tumor necrosis factor (TNF)-α or interleukin (IL)−6, neurotransmitters such as dopamine, GABA, or serotonin, and hormones regulate proliferation, maturation, and survival in adult neurogenic niches [12, 16].

Extrinsic factors are known to both positively and negatively impact adult neurogenesis. Stress and anxiety are consistent with significant reductions in proliferation and neuronal differentiation in neurogenic niches. Aging-related increased inflammation, neuroinflammation, and oxidative stress strongly inhibit neurogenesis. However, physical exercise and environmental stimuli can contribute to generating new neurons [14]. Dietary components such as flavonoids and essential vitamins and minerals promote neurogenesis, while high-fat and/or high-sugar diets harm the generation of new neurons [17]. Similarly, growing evidence reveals the influence of microbiota, particularly the microbiota-gut-brain axis, and dysbiosis on the activity of brain neurogenic niches [18].

### Brain renin-angiotensin system

RAS is classically considered a systemic/endocrine system involved in controlling blood pressure, cardiovascular homeostasis, and water and electrolyte balance regulation. In addition to the circulating RAS, there are local/paracrine RAS in many tissues, including the brain [19]. Additionally, an intracellular or intracrine RAS has been described, adding a third level of complexity to the system [20–23]. All components of RAS have been identified in the brain. Angiotensinogen is the precursor of all angiotensin (Ang) metabolites and is cleaved into Ang I by renin. Ang I is further processed by angiotensin-converting enzyme (ACE) to produce Ang II, which is the main neuroactive effector of RAS. Ang II binds to two main receptors: Ang II type 1 receptors (AT1R) and Ang II type 2 receptors (AT2R), with opposite effects. Aminopeptidases can also convert Ang II into Ang III, then Ang IV, which binds to the type II transmembrane-zinc protease insulin-regulated aminopeptidase (IRAP), initially identified as AT4 receptor (AT4R) [24]. Alternatively, Ang II can be converted into Ang (1–7) by ACE2, an isoform of ACE. Ang (1–7) binds to Mas receptors (MasR) with the highest affinity, although it can also bind to AT2R with low affinity. Ang (1–7) also binds to the more recently discovered receptor Mas-related-G-protein-coupled receptors (MRGPRs), whose main ligand is alamandine formed after decarboxylation of Ang (1–7) [25].

**Fig. 2:**
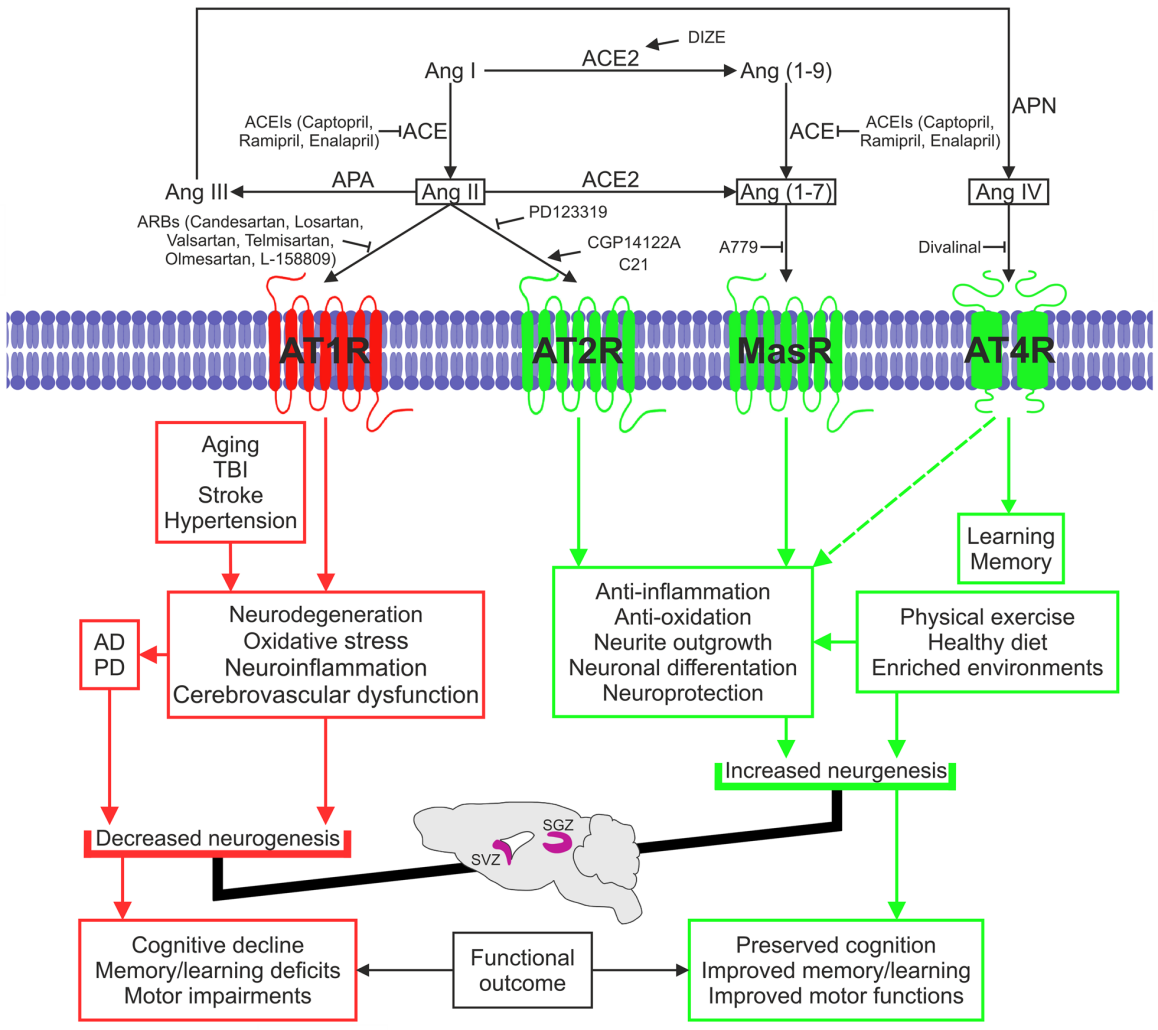
Effects of renin-angiotensin system (RAS) on adult neurogenesis. Ang: angiotensin; ACE: angiotensin-converting enzyme; ACEIs: angiotensin-converting enzyme inhibitors; ARBs: angiotensin receptor blockers; AT1R: angiotensin II type 1 receptor; AT2R: angiotensin II type 2 receptor; MasR: Mas receptor; AT4: angiotensin type 4 receptor; DIZE: diminazene aceturate; APA: aminopeptidase A; APN: aminopeptidase N

**Fig. 3:**
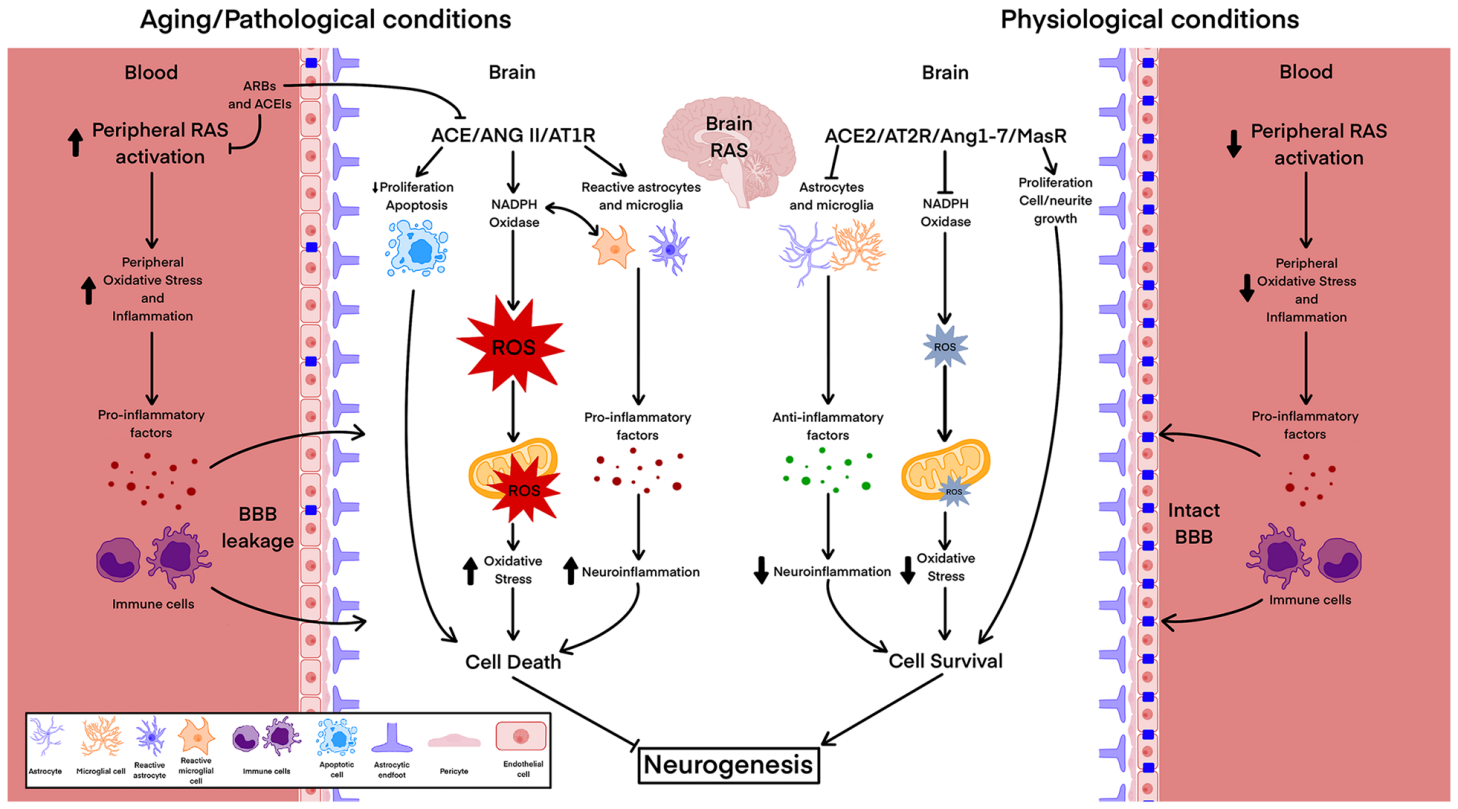
Mechanisms mediating the effects of peripheral and brain renin-angiotensin system (RAS) in adult neurogenesis. Ang: angiotensin; ACE: angiotensin-converting enzyme; ACEIs: angiotensin-converting enzyme inhibitors; ARBs: angiotensin receptor blockers; AT1R: angiotensin II type 1 receptor; AT2R: angiotensin II type 2 receptor; MasR: Mas receptor; BBB: blood–brain barrier

The current view considers the RAS as a system constituted by two arms: a classic arm, mainly formed by Ang II/AT1R, with pro-oxidative, pro-inflammatory, and pro-fibrotic effects, and a compensatory arm, which includes Ang II/AT2R and ACE2/Ang (1–7)/MasR, with anti-oxidative, anti-inflammatory and anti-fibrotic effects. Different studies showed that the overactivity of the Ang II/AT1R axis plays a major role in aging-related changes, immune and neuroinflammatory responses, microglial polarization, and neurodegeneration, while AT2R and MasR activation counterbalances these effects and functionally act as AT1 antagonists [26, 27]. In addition, RAS has also been involved in regulating cell proliferation, apoptosis, migration, and differentiation [28]. Ang II through AT1R promotes NSC proliferation and tissue growth [6, 29, 30]. AT2R is especially abundant in fetal tissues, suggesting a role during development. Previous works showed that Ang II via AT2R induced proliferation, neurite outgrowth, and neuronal differentiation from neural precursors and promoted neuronal migration. These effects were mediated by extracellular signal-regulated kinases (ERK) and mitogen-activated protein kinase (MAPK) pathways. However, phosphatidylinositol 3'-kinase (PI3K)/Akt inhibition failed to reverse Ang II-mediated NSC proliferation [7, 29, 31]. The interaction between AT2R and Wnt/β-catenin has also been suggested [32]. Furthermore, the expression of AT2R increases after cellular damage, suggesting a role in regeneration, probably through mechanisms involving pro-survival signals. However, other authors showed that activation of AT2R reduces cell proliferation [33] or has pro-apoptotic effects [34], suggesting that the effects of AT2R are highly dependent on the cellular environment and probably interact with other factors that impact their effects (see for review, [35]) (Fig. 2). Although the brain RAS is separated from the systemic RAS by the blood–brain barrier (BBB), circulating Ang II could also gain access to neural regions after leakage and increased BBB permeability during peripheral inflammatory processes, contributing to the feedback loops between both systems (Fig. 3).

## Role of RAS in adult neurogenesis under physiological conditions

### RAS components regulate adult neurogenesis

Different RAS components have been identified in neurogenic niches. The expression of AT1R and AT2R has been shown in NSCs derived from the embryonic and adult hippocampus [29, 36]. Distinct cell populations of the V-SVZ, including neuroblasts and GFAP-positive cells, a marker of NSCs and non-neurogenic astrocytes, also expressed AT1R and AT2R [37]. MasR or the enzyme ACE2 has been identified in rodent hippocampal areas [38–40]. Our group has recently demonstrated the involvement of RAS receptors in the regulation of rodent adult neurogenesis in the V-SVZ [37]. Activation of AT2R with the non-peptide agonist C21 increased the proliferation and generation of neuroblasts in the V-SVZ both in rats and mice. In accordance, reduced proliferation was observed in the SGZ of AT2R knock-out (KO) mice [41]. However, treatment with the AT1R antagonist candesartan promoted proliferation and neurogenesis in rats but not in mice, suggesting that the actions of RAS on adult neurogenesis may be mechanistically different depending on the species under study [37]. Conversely, other works showed that treatment with candesartan or losartan did not affect the number of Ki-67-positive cells (i.e., proliferating cells) and neuroblasts in the rat DG [42, 43]. Accordingly, systemic administration of Ang II did not significantly affect hippocampal neurogenesis in control mice [43]. Complex interactions between RAS receptors and differences in the AT1R/AT2R ratio may explain the controversial results obtained between species and neurogenic niches [37].

The role of other RAS components in adult neurogenesis has also been studied. Previous results showed that MasR deficiency did not significantly affect cell proliferation or cell death in the adult DG, although it induced an increase in the number of neuroblasts, suggesting a specific role in this population [44]. Pharmacological activation or blocking of MasR did not affect the generation of V-SVZ-derived neurospheres (i.e., floating aggregates formed by NSC/progenitor cells). However, treatment of V-SVZ-derived neurospheres with the MasR antagonist A779 blocked the increase in proliferation observed after stimulation of AT2R, suggesting a functional dependence between AT2R and MasR [37, 45]. Another RAS component expressed in the SGZ is the renin/prorenin receptor, also known as ATP6AP2 [46], which binds renin and prorenin, the inactive proenzyme form of renin, and leads to the production of Ang II. Co-labeling experiments have demonstrated that early mitotic cells in the hippocampus that are positive for Sox2 are negative for ATP6AP2. In contrast, young newly formed neurons that express DCX also express ATP6AP2 [47], hinting at its possible role in regulating adult neurogenesis. The downregulation of ATP6AP2 resulted in a marked reduction in the number of DCX-positive cells in the DG, and DCX-positive neuroblasts in ATP6AP2-KO mice resulted in abnormal morphology [48, 49]. Considering that disturbances in ATP6AP2 have been linked to intellectual disability in humans, investigations focusing on the role of ATP6AP2 could contribute to understanding different brain functions associated with neurogenesis.

### RAS mediates the effects of exercise on neurogenesis

Physical exercise is a robust stimulus that enhances hippocampal neurogenesis. Previous evidence suggested that RAS plays an important role in mediating the beneficial effects of physical activity on learning and memory. Exercise reduced hippocampal Ang II levels and AT1R expression, improving spatial memory and rescuing impaired neurogenesis in hypertensive mice [50]. Similarly, elevated systemic Ang II plasma levels, associated with worsening cognition, were normalized after exercise in heart failure and hypertensive patients [51]. This evidence contrasts with other findings showing no changes [52] or increased levels of Ang II in plasma after exercise [53] and that systemic administration of Ang II increased proliferation and neurogenesis in the DG. This effect was abolished by the AT1R antagonist losartan [43]. These studies were conducted with healthy controls, while the results mentioned above were obtained in pathological conditions, in which plasma levels of Ang II are elevated. The type and intensity of physical activity and sex could also impact the results [54, 55].

It is well-known that trophic factors such as BDNF and VEGF are involved in the running-enhanced neurogenesis in the DG [56, 57]. Previous data showed complex, bidirectional interactions between trophic factors and Ang II signaling [58]. RAS also exerts modulatory actions on neurotransmitters [5, 59, 60], which play an important role in neuronal survival, neuroplasticity, and neurogenesis [61]. Previous studies showed reduced brain serotonin levels in ACE2-KO mice. Although no changes in baseline SGZ neurogenesis were observed in these animals, ACE2 deficiency impaired running-induced neurogenic response in the DG, while MasR deficiency had no significant effects [62]. These results point out ACE2 as a novel factor required for exercise-dependent modulation of adult neurogenesis. This is in line with evidence showing that exercise induces ACE/Ang II/AT1R axis downregulation and ACE2/Ang (1–7)/MasR axis upregulation [63], an effect that may depend on the exercise protocol [64].

### RAS modulates aging-related impaired neurogenesis

Previous data showed aging-associated dysregulation of RAS, particularly the overactivity of the Ang II/AT1R axis [65–67]. Similarly, aging is associated with structural changes in the neurogenic areas, a reduced number of NSCs, and alterations in multiple steps of adult neurogenesis. Previous studies suggest the relationship between RAS signaling and different key factors in the regulation of neurogenesis, including Wnt/β-catenin and Notch pathways, growth factors such as BDNF, and cytokines such as TNF-α and IL-6 [68–70]. Interestingly, we have shown that the reduction observed in proliferation and neurogenesis in aged rodents can be counteracted by treatment with AT2 agonists or AT1 antagonists, suggesting RAS-modulating drugs as potential tools to mitigate aging-related neurogenesis impairment [37]. Considering that aging is a major risk factor for multiple diseases in which neurogenesis is affected and the imbalance of RAS with age, we will focus on the role of RAS as a possible strategy to preserve adult neurogenesis in different pathological conditions.

## Role of RAS in adult neurogenesis under pathological conditions

### Hypertension

Hypertension is one of the major risk factors for central nervous system disorders such as cerebrovascular diseases, neurodegenerative diseases, or age-related cognitive deficits, and dysregulation of RAS has a pivotal role in the pathogenesis of hypertension. Increased levels of circulating Ang II associated with hypertension alter BBB integrity, facilitating access of systemic Ang II, inflammatory factors, and immune cells to the brain. These factors contribute to brain RAS activation, with a consequent increase in neuroinflammation and oxidative stress, which may harm neurogenesis [71] (Fig. 3). Recent studies have shown robust glial activation, increased levels of pro-inflammatory cytokines, reduced number of neuroblasts in the hippocampus, and impaired hippocampus-related memory function in murine models of hypertension [50, 72]. Treadmill exercise can restore neurogenesis and ameliorate spatial memory function in hypertensive animals by reducing elevated systemic Ang II levels in plasma, counteracting the increase in the expression of AT1R in the hippocampus, and preventing the hippocampal BBB leakage [50]. Interestingly, AT1R blockade with candesartan can also increase hippocampal proliferation and neurogenesis by reducing NADPH oxidase (Nox) activation and reactive oxygen species (ROS) production by suppressing MAPK and NFκB and activating Wnt/β-catenin signaling. Moreover, treatment with angiotensin receptor blockers (ARBs), such as candesartan and losartan, enhanced neurogenic modulators and decreased the number of apoptotic cells in the hippocampus, suggesting increased cell survival. AT1R blockade with losartan also reduced the levels of proinflammatory markers such as TNF-α, IL-1β, and IL-6, while hypoxia markers such as hypoxia-inducible factor (HIF)-1α remain unchanged [68]. Interestingly, ARB effects were independent of their blood pressure-lowering action [72, 73]. In line with this, previous findings showed that Ang II reduced proliferation and promoted apoptosis in hippocampal-derived NSCs. After blocking AT1R with candesartan or losartan, mitochondrial ROS increased through AMPK-PGC1α-Bax signaling, improving learning and memory in models of chronic hypertension and hypertensive heart failure [74]. Previous results showed that a combination of ACE inhibitor (ACEI) captopril and atorvastatin reduced the impairment in the extinction of aversive memories, accompanied by increased hippocampal neurogenesis, in middle-aged mice [75]. Considering that hypertension and hyperlipidemia are modifiable risk factors for cognitive decline and an important percentage of adults over age 65 use both anti-hypertensives and statins to treat these conditions, combined therapies would represent a promising therapeutic option. Clinical trials showed that treatment with ARBs was associated with a lower risk of progression to dementia compared with ACEIs in patients with hypertension and mild cognitive impairment [76]. Treatment with ARBs but not ACEIs showed protective effects on cognitive functions in Parkinson’s disease (PD) patients with hypertension compared to other antihypertensive drugs [77]. However, their possible effects on neurogenesis have not been addressed.

The role of ACE2/Ang (1–7)/MasR has been recently studied in a rat model of hypertension. Treatment with the ACE2 activator diminazene aceturate (DIZE) reduced hippocampal glial activation and Ang II levels, along with a reduction in NOX4-induced oxidative stress, TRAF6-NFкB-induced inflammation, and mitochondrial dysfunction, while increasing Ang (1–7) levels and MasR expression. ACE2 activation also promoted the proliferation and generation of new neurons in the hippocampus by activating Wnt/β-catenin signaling, which plays a crucial role in neurogenesis. These results suggest a dual effect of ACE2 activation on preventing neuroinflammation while promoting neurogenesis in hypertension [70, 78].

### Effects of RAS modulation on neurogenesis in cognitive dysfunction and dementia

Around 55 million people worldwide have dementia, which makes this condition a major health concern. Hypertension is one of the main modifiable risk factors for all-cause cognitive decline and dementia. Preclinical evidence suggests that treatment with antihypertensive medication, and particularly ARBs, reduces the risk of dementia (see for review [79]). Similarly, clinical and cohort studies suggested that treatment with antihypertensive drugs was associated with a lower risk of dementia, even in elderly and frail patients [80, 81]. We will discuss below the role of RAS in different conditions associated with dementia and cognitive impairment.

#### Alzheimer’s disease

Several studies have shown an imbalance of RAS in AD, particularly overactivation of ACE/Ang II pathway, suppressed ACE2/Ang (1–7) pathway, and dysbalanced AT1R and AT2R expression in the brain [82, 83]. Treatment with ARBs such as losartan, valsartan, telmisartan, and olmesartan, ACEIs such as captopril, or activation of the counter-regulatory RAS arm has shown promising results in different rodent models of AD, improving cerebrovascular function and rescuing memory [84, 85]. However, a few works addressed the impact of RAS manipulation on neurogenesis in cognitive impairment. Treatment with losartan increased the expression of neurogenic markers and restored cognitive deficits by reducing oxidative stress and inflammation [73], with a slight increase in the number of 5-bromo-2’-deoxyuridine (BrdU)-positive cells in the DG of AD animal models [86]. These effects were independent of changes in amyloidosis and blood pressure and were counteracted by treatment with the AT2R antagonist PD123319. However, AT2R agonism using C21 failed to recover memory and exerted limited benefits on vascular function and inflammation. Treatment with the AT4R antagonist divalinal counteracted losartan’s benefits, while Ang IV increased hippocampal cellular proliferation and dendritic arborization, reduced oxidative stress, and restored short-term memory, spatial learning, and memory [86–88], also suggesting the involvement of the Ang IV/AT4R cascade. Other studies showed that treatment with candesartan in AD transgenic mice increased hippocampal proliferation without changes in the population of neuroblasts, despite normalizing the length of their dendritic arborization [89]. Although these results point to the limits of candesartan in rescuing memory in AD mice, alternative explanations could be the dose used, the treatment period, the stage of the disease, or that the peroxisome proliferator-activated receptor (PPAR)-γ agonist property of candesartan does not confer an added value in treating dementia [89]. Clinical studies have also shown that antihypertensive drug treatments, including ARBs and ACEIs, are associated with reduced cognitive decline and dementia [90, 91]. Although comparative studies between ARBs and ACEIs are limited, the results suggest greater benefits associated with ARB use than ACEIs, with less brain atrophy, slower cognitive decline, lower dementia, and fewer plaques and tangles [92]. Aspects such as the ability of these drugs to penetrate the BBB, larger cumulative dose or longer duration, and other patient-related factors, such as genetic variances, could contribute to the heterogeneity of the results [93–95].

#### Vascular dementia and ischemic brain injury

Vascular dementia is a common type of dementia caused by reduced blood flow to the brain. Cognitive functions and neurogenesis are significantly impaired in animal models of vascular dementia. Previous data showed preserved cognition and neurogenesis in MasR-deficient mice despite reduced cerebral blood flow. However, double AT2R/MasR-KO mice showed marked cognitive deficits and a tendency towards reduced neurogenesis in the DG [45]. Therefore, MasR deficiency could benefit cognitive function in vascular cognitive impairment when AT2 is preserved. Functional interactions and heterodimerization between AT2R and MasR and/or RAS imbalance after MasR deletion, which provides beneficial effects indirectly, could explain these effects [37, 96].

Ischemic brain injury is often associated with functional and cognitive deficits. Considering the role of RAS in neurodegeneration, previous works addressed the possible implication of RAS in cognitive impairment in this neurodegenerative scenario. Cognitive impairment observed after focal brain injury induced by middle cerebral artery occlusion was significantly higher in AT2-KO mice compared to age-matched wild-type (WT) animals. Treatment with the AT1R antagonist valsartan prevented cognitive decline in WT mice, in a process mediated by methyl methanesulfonate sensitive 2 (MMS2), which has neuroprotective properties and an important role in DNA repair through the ubiquitin–proteasome system. However, the effect of valsartan was weaker in AT2-KO mice. Treatment with Ang II and valsartan increased differentiation into neuron-like cells in culture, which was inhibited by AT2R antagonism [97], suggesting a pivotal role of AT2R even in control conditions. Sex differences in the role of AT2R have been reported [98]. Female AT2-KO mice showed significantly decreased hippocampal neurogenesis and impaired cognitive function after stroke compared to AT2-KO males. However, male AT2-KO mice showed higher AT1R expression, accompanied by larger ischemic brain damage and decreased cerebral blood flow than females. These results suggest that sex differences may depend, at least in part, on the balance between AT1R and AT2R expression, and point to the need for further gender-sensitive research [37, 41].

#### Parkinson’s disease

PD is a neurodegenerative disorder characterized by motor disturbances resulting from the degeneration of dopaminergic neurons, although most late-stage PD patients suffer from cognitive deficits and dementia [99]. The most widely accepted view states that neurogenesis is affected in PD after dopaminergic depletion, accumulation of misfolded proteins, and/or signals associated with neurodegeneration/neuroinflammation, leading to abnormal brain plasticity and motor and cognitive impairments. Our group has shown that the reduction in the V-SVZ neurogenesis observed in animal models of PD can be restored after inhibition of AT1R or activation of AT2R, revealing interactions between dopamine and RAS in this niche, and pointing out the potential beneficial effects of RAS modulators on the regulation of neurogenic capacity in neurodegenerative diseases [16]. Note that recent studies showed a reduced risk of PD in patients with hypertension or ischemic heart disease treated with RAS inhibitors, with a higher cumulative duration of BBB-crossing ARBs [4, 100, 101]. A recent clinical study also suggested that ARB-treated patients had lower anxiety scores than those treated with ACEIs [102]. This provides insight into the development of disease-modifying drug candidates for PD, adding to existing evidence.

#### Brain irradiation

Partial or whole-brain irradiation as a treatment for brain tumors or metastasis is frequently associated with cognitive deficits related to hippocampal neurogenesis impairment [103]. Neuroinflammation and microglial activation have been suggested as mechanisms involved in cognitive dysfunction induced by brain irradiation. Therefore, targeting the RAS with modulators possessing anti-inflammatory activity could provide neuroprotection and mitigate these effects. Treatment with AT1R antagonist L-158809 administered before, during, and after whole-brain irradiation ameliorates hippocampal-dependent cognitive tasks short-term (i.e., 24 h to 12 weeks) and long-term (i.e., up to 12 months) in young male rats [104–106]. However, AT1R blockade did not affect microglial proliferation/activation, pro-inflammatory cytokine levels, or proliferation and neurogenesis in the DG [105]. In contrast, these authors observed reduced irradiation-induced inflammation in peripheral tissues after L-158809 treatment [107, 108]. These controversial results could be explained by the dose and timing range of treatment or the route of administration (i.e., oral treatment) since there is a lack of evidence on the ability of L-158809 to cross the BBB. Chronic administration of the ACEI ramipril after a single 10 Gy dose of radiation had a small but significant effect in mitigating the deleterious effects on progenitor proliferation and neuronal differentiation in the rat DG by reducing apoptosis in SGZ progenitors [109]. However, ramipril had no significant effect on the reduction of neurogenesis observed after exposure to higher doses of radiation (i.e., 15 Gy) [109] or fractionated whole-brain radiation (i.e., a 40 Gy total dose) [110], possibly due to differences in the timing and dose of ramipril, and/or the degree of neuroinflammation that occurs after different doses of X-radiation. Unlike losartan [43], ramipril suppresses basal neurogenesis in the DG in control animals that received sham radiation [109]. Different mechanisms of action could explain this since ramipril inhibits AT1R and AT2R, while losartan antagonizes AT1R, leading to an increase in AT2R expression. Alternatively, the effects of ramipril could also result from a long-term treatment or the relatively high dose of ramipril used in the study, which was approximately twice the standard clinical dose [109]. Strategies that combine ramipril and atorvastatin (i.e., a statin that blocks the rate-limiting cholesterol biosynthesis) have also been tested, and a synergistic beneficial effect resulting in a 2.5-fold increase in neurogenesis was observed compared to irradiated animals treated only with atorvastatin [111]. However, it is unknown whether this increase in neurogenesis translates into improvements in cognitive function, suggesting the need for further studies [112].

Previous data showed a correlation between the expression of RAS components in patient-derived glioblastoma cells, the most prevalent and aggressive primary brain cancer, and tumor microenvironmental phenotype, response to standard chemoradiotherapy, and survival outcomes [113, 114]. Ang II/AT1R expression is associated with tumor growth and a more aggressive phenotype. Chemoradiation treatment upregulated the expression of *AGTR1* and *ATP6AP2* genes, and increased expression of stem cell markers, associated with a more aggressive subtype [115]. AT1R inhibition with losartan and telmisartan, but not valsartan, and the blocking of prorenin receptors reduced cell growth, proliferation, stemness, and angiogenesis in glioblastoma cells [114, 116–118], suggesting that the efficacy of some ARBs may also depend on pharmacological off-target effects [116]. In contrast, other studies showed reduced proliferation after AT2R inhibition, but no effects were observed after treatment with the AT1R antagonist losartan [119]. While these results were obtained under nutrient starvation and RAS receptor expression at biologically relevant levels, the studies mentioned above were conducted under RAS receptor overexpression conditions, which could disturb the equilibrium between the multiple components of the RAS. A phase I, open-label, proof-of-concept trial of RAS modulators in recurrent glioblastomas showed promising results with increased overall survival [120]. These data suggest modulation of RAS as a target for the prevention of cognitive impairment after brain irradiation and better outcomes in glioblastoma patients.

### Effects of RAS modulation on neurogenesis in traumatic brain injury

Studies in rodents suggest that traumatic brain injury (TBI) frequently induces morphological and physiological abnormalities in the adult hippocampus [121]. TBI is strongly associated with brain inflammation, and blocking AT1R has shown reduced microglial response and improved neurological outcomes [122, 123]. Intracerebroventricular administration of the AT2R agonist CGP14112A had neuroprotective effects, reduced the lesion volume, and enhanced neurogenesis in the DG and the V-SVZ by increasing the number of proliferative cells and newly generated neuroblasts [124]. Upregulation of BDNF and neural growth factor (NGF) and the phosphorylation of Akt and ERK1/2, followed by HIF-1α induction, were involved in these effects. Akt phosphorylation increases nitric oxide levels, improving the blood flow, while ERK1/phosphorylation promotes neuronal differentiation and neurite outgrowth [125]. Increased levels of BDNF also upregulate AT2R, which are involved in axonal plasticity and outgrowth [126]. Interestingly, AT2R activation also improved motor and cognitive function after TBI. However, these benefits showed a delayed onset, possibly because of the timeframe needed for the new cells to become integrated [124]. Blocking AT2R reduced the neuroprotective effects and increased neurogenesis observed after preconditioning by exposure to heat acclimation in TBI animal models, suggesting again that AT2R acts as a modulator of neurorepair [127]. Controversial results showed increased proliferation of NSC/progenitor cells and increased newly generated cells in the V-SVZ and DG after TBI, probably because of compensatory effects [128, 129]. Recent studies suggest that this initial increase in neurogenesis could have adverse effects by exhausting neurogenic potential. Therefore, treatments focused on slowing down the increase in neurogenesis can potentially restore neurogenic capacity and minimize side effects.

Other studies showed that treatment with the ACEI captopril or enalapril before TBI resulted in increased signs of neurodegeneration and exacerbated motor deficits in male rats compared to control animals. Increased levels of substance P, which mediate alternative routes of programmed cell death and inflammatory processes, and/or the ability of centrally acting ACEIs to increase bradykinin, which has pro-inflammatory properties, could explain these effects [130]. However, there is no conclusive data on the effect of these drugs on neurogenesis in patients affected by TBI. Some recent results suggest that ACEIs exacerbate brain damage and worsen functional outcomes in patients with isolated TBI [131]. Combinations of ACEIs and statins significantly lowered the risk of dementia and possible AD in patients with a TBI history [132]. Therefore, the convergence of different factors/treatments or the influence of genetic factors such as variations in specific regions of ACE genes should be considered in TBI patients (see for review, [133]).

## Limitations and future perspectives

Numerous preclinical studies suggest the potential effectiveness of RAS inhibitors as a therapeutic strategy to preserve neurogenesis and cognitive functions in aging and different pathological conditions. Similarly, some clinical studies, mainly observational studies, report benefits. However, there is still contradictory evidence, possibly associated with the study designs, animal models/patient characteristics, drugs, and regimens, resulting in high heterogeneity, which makes it difficult to conclude. Understanding and accounting for interspecies differences is essential for improving the translational value of preclinical research. Failure to consider these differences may limit the clinical predictability of experimental findings. Another point to consider is the poor permeability of some RAS modulators across the BBB, which could hamper their effects. The use of prodrugs and new strategies for enhancing brain targeting, such as intranasal delivery or nanoparticle-based strategies, could contribute to overcoming this limitation [134, 135]. A relevant aspect relates to the gender dimension. There is evidence for the influence of sex on RAS signalling. Numerous studies have also reported sex-linked differences in neurogenesis. However, hardly any studies address the role of RAS in neurogenesis considering both sexes. The design of new preclinical and clinical studies considering these factors is essential. Given that current pharmaceutical treatments to preserve neurogenesis have only shown modest effects on symptoms derived from its decline, repurposing existing medications, such as ARBs and ACEIs, and modifying risk factors are promising lines of research that should be explored in depth.

## Conclusion

Alterations in neurogenesis occur concurrently with or may contribute to the development of different pathological conditions. Identifying new targets and strategies to preserve adult neurogenesis and tackle the disabling effects associated with alterations in neurogenesis becomes a prime health concern. There is evidence of the role of RAS in regulating adult neurogenesis in both physiological and pathological conditions (Fig. 2). Although several mechanisms by which RAS modulates adult neurogenesis have been identified (Fig. 3), there are controversial data, and several aspects remain unknown or unconfirmed. An important advantage is that RAS modulators such as ARBs and ACEIs are already clinically available, and repositioning these drugs to preserve or restore neurogenesis is an attractive option. This review summarizes evidence on the capacity of RAS modulators, commonly used to treat high blood pressure, to promote neurogenesis and aims to pave the way for their use in the clinic to prevent or reduce the onset of some of the symptoms of neurogenesis decline, such as cognitive impairment and dementia. However, not all ARBs and ACEIs seem to have the same effects and/or efficacy in preventing neurogenesis decline and its consequences. Other factors influencing effectiveness, such as timing, dosages, permeability across the BBB, and the therapeutic window, may also be considered. Identifying the most appropriate high-risk groups and defining the profile of patients best suited to respond to these treatments remain challenging. Therefore, it is essential to deeply investigate the most effective ARBs and ACEIs and understand the underlying mechanisms to obtain robust results. Considering different contributory factors such as sex, aging, and comorbidities is crucial to developing more effective and personalized treatments with reduced side effects. There is a clear need for long-term follow-up and well-powered randomized clinical trials that evaluate neurogenesis, memory, and cognitive outcomes as primary endpoints, which will lay the groundwork for new therapeutic options in this area.

## Data Availability

Not applicable.

## Abbreviations

ACE: Angiotensin-converting enzyme
ACEI: Angiotensin-converting enzyme inhibitor
AD: Alzheimer’s disease
Ang: Angiotensin
ARB: Angiotensin receptor blocker
AT1R: Angiotensin II type 1 receptor
AT2R: Angiotensin II type 2 receptor
AT4R: Angiotensin II type 4 receptor
BBB: Blood–brain barrier
BDNF: Brain-derived neurotrophic factor
BrdU: 5-Bromo-2’-deoxyuridine
DCX: Doublecortin
DG: Dentate gyrus
DIZE: Diminazene aceturate
EGF: Epidermal growth factor
ERK: Extracellular signal-regulated kinase
FGF-2: Fibroblast growth factor-2
GFAP: Glial fibrillary acidic protein
HIF-1α: Hypoxia-inducible factor-1α
IGF-1: Insulin growth factor-1
IL-6: Interleukin-6
IRAP: Type II transmembrane-zinc protease insulin-regulated aminopeptidase
KO: Knock-out
MAPK: Mitogen-activated protein kinase
MasR: Mas receptor
MMS2: Methyl methanesulfonate sensitive 2
MRGPR: Mas-related-G-protein-coupled receptor
Nox: NADPH oxidase
NSC: Neural stem cell
PD: Parkinson’s disease
PI3K: Phosphatidylinositol 3'-kinase
PPAR-γ: Peroxisome proliferator-activated receptor-γ
PSA-NCAM: Polysialylated neural-cell-adhesion molecule
RAS: Renin-angiotensin system
ROS: Reactive oxygen species
SGZ: Subgranular zone
TBI: Traumatic brain injury
TNF-α: Tumor necrosis factor-α
VEGF: Vascular endothelial growth factor
V-SVZ: Ventricular-subventricular zone
WT: Wild type
